# *Ginkgo biloba* Extract Prescriptions Are Associated with Slower Progression of Dementia Severity—Analysis of Longitudinal Real-World Data

**DOI:** 10.3390/brainsci15010012

**Published:** 2024-12-26

**Authors:** Jens Bohlken, André Hajek, Martin Burkart, Karel Kostev

**Affiliations:** 1 Institute of Social Medicine, Occupational Health and Public Health, Faculty of Medicine, University of Leipzig, 04103 Leipzig, Germany; 2 Department of Health Economics and Health Services Research, University Medical Center, 20246 Hamburg, Germany; 3 Global Medical Affairs, Dr. Schwabe Holding SE & Co. KG, 76227 Karlsruhe, Germany; 4 Epidemiology, IQVIA, 60549 Frankfurt am Main, Germany

**Keywords:** dementia, Alzheimer’s disease, *Ginkgo biloba* extract, cognitive impairment, phytomedicine, retrospective study

## Abstract

Background/Objectives: Previous research indicates that *Ginkgo biloba* extract (Gbe) may contribute to slowing down the progression of dementia. This retrospective cohort study analyzed the association between Gbe prescriptions and the progression of dementia severity in a real-world setting. Methods: This study was conducted using data from patients with an initial diagnosis of mild or moderate dementia between January 2005 and December 2022 from the IQVIA™ Disease Analyzer database. The follow-up period was up to 10 years. The association between Gbe prescription and dementia severity progression was assessed by Kaplan–Meier analysis and Cox regression, with adjustments made for age, sex, health insurance status, physician specialty, co-prescription of antidementia drugs, and co-diagnoses. Results: A total of 4765 patients were included, of whom 177 were prescribed Gbe. The cumulative incidence of dementia severity progression was 12.7% for patients with Gbe prescription and 22.1% for those without. Gbe prescription was associated with a significantly decreased risk of dementia severity progression (hazard ratio: 0.50; 95% CI: 0.27–0.95), both in patients with mild (HR: 0.44; 95% CI: 0.22–0.90) and moderate dementia (HR: 0.24; 95% CI: 0.06–0.98). Conclusions: This study provides evidence that Gbe prescription is associated with a reduced risk of dementia severity progression in a real-world setting.

## 1. Introduction

Dementia is defined by the acquisition of cognitive impairment, which is predominantly caused by vascular disorders or progressive neurodegenerative diseases. The most common types include Alzheimer’s disease, Lewy body dementia, and frontotemporal degeneration [1]. Most frequently mixed pathologies are identified at autopsy, and vascular alterations are the most frequent pathologies. Each type has unique risk factors, such as age, genetics, cardiovascular health, comorbidities, environmental factors, and lifestyle choices [1].

The disease typically progresses from mild cognitive impairment (MCI), which is defined as objective cognitive deficits but preserved independent living. Thus, there is a continuous progression from a healthy state to mild, moderate, or severe impairment, eventually resulting in an individual’s inability to perform daily activities and live independently [1]. The progression of dementia varies significantly. While vascular pathology can be characterized by stepwise deterioration and phases of stability, neurodegenerative disorders often show continuous gradual decline. Clinical progression of neurodegenerative disorders can also be very heterogeneous, both within and between patients [2]. Some patients exhibit rapid decline while others progress more slowly. One potential underlying pathomechanism is the altered production and clearance of AD-related proteins, which contributes to an imbalance that drives AD progression [3].

In 2021, approximately 1.8 million people in Germany were living with dementia, with a projected number of up to 2 million in 2033 [4]. Globally, 47 million people or 5% of the world’s elderly population were affected by dementia in 2015. By 2030, this number is predicted to increase to 75 million [5]. For the treatment of patients with mild to moderate dementia, national and international guidelines recommend the use of *Ginkgo biloba* extract (Gbe) EGb 761 (EGb 761^®^ is the proprietary extract and active ingredient in pharmaceuticals manufactured by Dr. Willmar Schwabe, Karlsruhe, Germany), such as the German S3-Guidelines [6] or the international guidelines of the World Federation of Societies of Biological Psychiatry (WFSBP) [7]. EGb 761 is a dry extract from *Ginkgo biloba* leaves (drug–extract ratio 35–67:1), approved as medicinal products in numerous countries worldwide (with brand names such as Tebonin^®^ or Tanakan^®^), and complies with the specifications of the European Pharmacopoeia (Ph. Eur.) monograph. The extract must contain 22.0–27.0% Ginkgo flavonoids, calculated as Ginkgo flavone glycosides and 5.4–6.6% terpene lactones consisting of 2.8–3.4% ginkgolides A, B, C, and 2.6–3.2% bilobalide and less than 5 ppm of ginkgolic acids.

In systematic reviews of clinical trials, beneficial effects of EGb 761 in patients with MCI and dementia were seen in several domains of cognition such as memory, speed of processing, attention, and executive functioning [8,9]. In the treatment of mild to moderate dementia, EGb 761 has been demonstrated to be superior to placebo concerning cognitive function, activities of daily living, and global status over 22 to 26 weeks of treatment [10]. Wettstein [11] calculated that EGb 761 can delay dementia progression by approximately seven to eight months. The evaluation was based on effect sizes on cognition, as measured by the Alzheimer’s Disease Assessment Scale cognitive subscale (ADAS-cog), in placebo-controlled trials related to the cognitive decline in the placebo groups. As patients treated in dementia trials often improve initially, the time to return to the baseline score was compared between EGb 761 and placebo in a sensitivity analysis. A previous study analyzing the impact of Gbe drug prescriptions on progression from MCI to dementia in a real-world setting found that all-cause dementia incidence decreased with higher numbers of Gbe prescriptions [12]. The plant extract displays various pharmacodynamic effects. A review identified reduction in neuroinflammation, increase in neuroplasticity, decrease in formation of hyperphosphorylated tau proteins and amyloid protein aggregation, increase in neurotransmission, and improvement of microcirculation as the main pharmacodynamic effects [13]. However, data from long-term clinical studies on dementia progression are not available so far.

The aim of this retrospective cohort study was to analyze, for the first time, the long-term impact of *Ginkgo biloba* on the progression of dementia based on a large dataset of qualitative information on degrees of severity based on the clinical judgement of physicians. The data were derived from the same database as used before [12].

## 2. Materials and Methods

### 2.1. Data Source and Study Design

This retrospective cohort study used the IQVIA™ Disease Analyzer database, which contains information on patient consultations from Germany. The database has been sourced from nearly 3000 office-based general practitioners and specialists, representing approximately 3–5% of all German practices (IMS^®^DA status date: March 2024). The database contains datasets of more than ten million patients, comprising demographic information, diagnoses, drug prescriptions, sick leave, and referrals to hospitals from a period of more than 15 years (2005 to 2023). The sample of practices encompasses eight major regions and is geographically representative of the entire country of Germany. Regarding the incidence and prevalence of major chronic diseases, the database exhibited a high degree of concordance with national reference data from official sources, suggesting that it is representative and valid. Therefore, the database is considered suitable for pharmaco-epidemiological studies [14]. The IQVIA™ Disease Analyzer database has previously been successfully employed in the context of dementia-related research [15,16].

### 2.2. Study Population

The study population included individuals in outpatient care in Germany (general practitioners, neurologists, or psychiatrists) with a first diagnosis of dementia (ICD-10: F00, F01, F03, G30) in the period from January 2005 to December 2022 and with documentation of mild or moderate severity in the electronic medical record. Only data from practices with documentation of dementia severity were used (76 GPs and 47 neurologists or psychiatrists). Since the severity of dementia is not encoded in an International Classification of Diseases (ICD) code, the information was extracted from free text information based on the clinical judgement of the physicians.

Patient data were evaluated if follow-up information were available after the index date, defined as the day of the initial dementia diagnosis in the medical record. The following exclusion criteria were applied: (1) patients who had been prescribed Gbe or antidementia drugs (memantine, rivastigmine, donepezil, galantamine) prior to the index date, (2) age < 60 years at index date, and (3) diagnosis of severe or moderate dementia prior to the diagnosis of mild dementia or diagnosis of severe dementia prior to moderate dementia. Each patient was followed from the index date until the timepoint at which a more severe stage of dementia was documented, the last consultation occurred, or the end of the 10-year follow-up period was reached, whichever occurred first.

The following types of progression were evaluated: change from mild dementia to moderate dementia, from mild dementia to severe dementia, and from moderate dementia to severe dementia. The prescriptions issued between the index date and the subsequent dementia stage or the conclusion of the follow-up period for each patient were evaluated to ascertain whether they included a Gbe product. The latter was defined as a medicinal product manufactured in accordance with the standards set forth in the European Pharmacopoeia. Consequently, homeopathic medicines and dietary supplements were not considered.

### 2.3. Statistical Analysis

The cumulative incidence of progression from mild dementia to moderate or severe dementia and of progression to severe dementia in patients with moderate dementia was analyzed using Kaplan–Meier survival analysis and displayed using Kaplan–Meier curves. In each analysis, patients with Gbe prescriptions were compared with patients without Gbe prescription. Differences between cumulative incidence of Gbe and non-Gbe patients were compared using log-rank-tests.

The association between Gbe prescription and risk of dementia progression was additionally assessed using Cox regression adjusted for age, sex, health insurance status, physician specialty, co-prescription of antidementia drugs, and co-diagnoses. Analysis of comorbidities was based on ICD-10 coded clinical diagnoses entered by the treating physicians into the electronic medical records in routine clinical practice. These variables were selected as they may be associated with both dementia risk and prescription of Gbe. Results of the Cox regression model are displayed as hazard ratios (HRs) and 95% confidence intervals (CIs). A *p*-value of <0.05 was considered statistically significant. Analyses were conducted using SAS version 9.4 (SAS Institute, Cary, NC, USA).

## 3. Results

### 3.1. Baseline Characteristics

**Figure 1:**
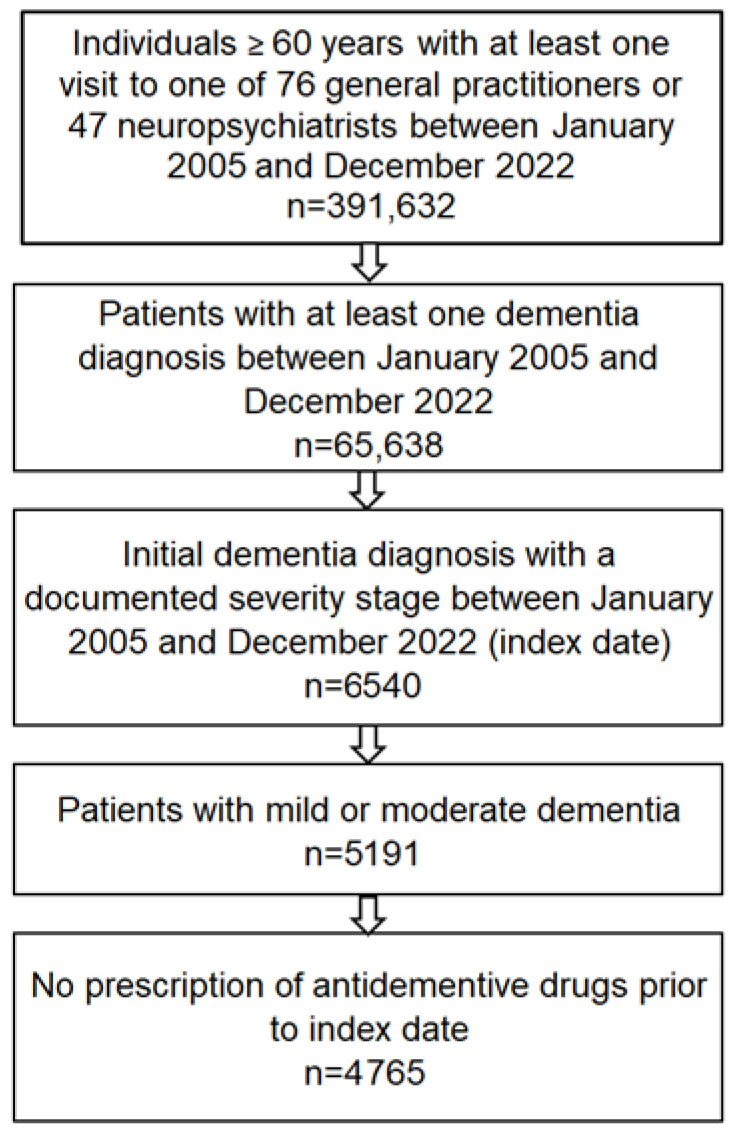
Selection of patients.

The selection of patients resulted in data from 4765 patients to be analyzed (Figure 1). Table 1 shows the baseline characteristics of the included patients. The average age was 79.6 (SD: 6.5) years, and 63.0% were female. Mild dementia was documented for 42.5% of patients and moderate dementia for 57.5%; 63.9% of patients received treatment from a neurologist or psychiatrist, while 36.1% received treatment from a general practitioner. The proportion of privately insured patients was 13.1%.

### 3.2. Prevalence of Gbe Prescription

In total, 177 patients (3.7%) received at least one prescription of Gbe. Table 2 shows the frequency in relation to patient characteristics. The proportion of Gbe prescription was higher in privately insured (7.7%) than in statutory insured (3.1%) patients and higher in general practices (7.2%) than in practices of neurologists or psychiatrists (1.8%). No relevant differences were observed between age groups, women and men, or in patients with either mild or moderate dementia.

### 3.3. Cumulative Incidence of Dementia Progression

**Figure 2:**
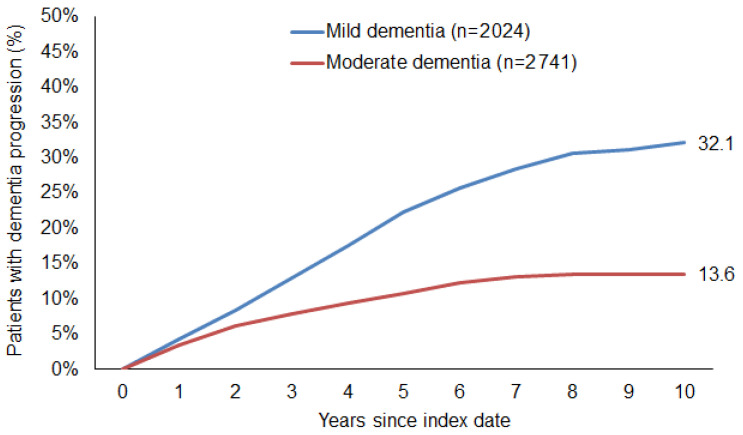
Cumulative incidence of dementia progression in patients with mild and moderate dementia.

**Figure 3:**
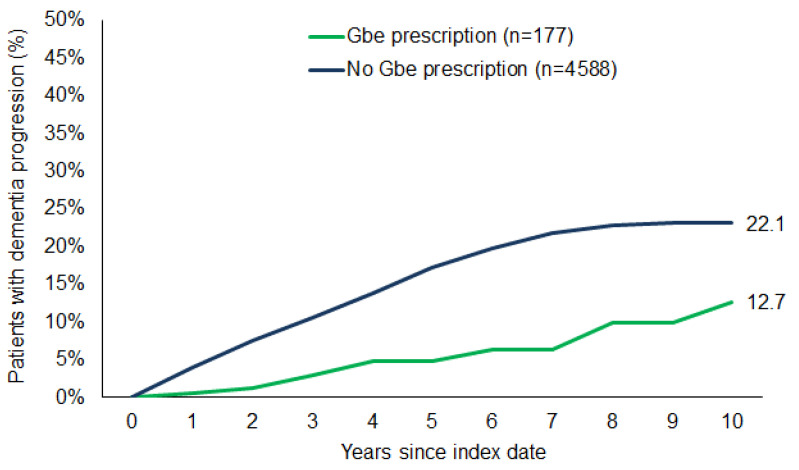
Cumulative incidence of dementia progression in patients with and without Gbe prescription.

In the follow-up period of up to ten years after the index date, the estimated cumulative incidence of dementia severity progression was 32.1% in patients with mild dementia and 13.6% in patients with moderate dementia (log-rank-test *p* < 0.001; Figure 2). The cumulative incidence of severity progression was 12.7% in patients with Gbe prescription versus 22.1% in those without (log-rank-test *p* < 0.001; Figure 3).

### 3.4. Association Between Gbe Prescription and Dementia Severity Progression

In a multivariate Cox regression model, the prescription of Gbe was found to be associated with a significantly decreased risk of dementia severity progression (HR: 0.50; 95% CI: 0.26–0.95). This association was significant in both patients with mild dementia (HR: 0.44; 95% CI: 0.22–0.90) and patients with moderate dementia (HR: 0.24; 95% CI: 0.06–0.98) (Table 3).

## 4. Discussion

In this retrospective study, Gbe prescriptions were associated with a significantly lower risk of dementia severity progression. Stratification by baseline severity dementia revealed that this association was present in both mild and moderate stages. These findings corroborate the results of a meta-analysis on a specific Gbe, EGb 761, that demonstrated beneficial effects across different stages of dementia. Th extract stabilized cognitive performance and social functioning in both severity groups [10].

Our results are in accordance with published research suggesting that the effect of EGb 761 could delay the symptom progression in AD by approximately 8 months [17]. A delay of dementia progression in patients with AD treated with EGb 761 has also been reported in other real-world datasets. Compared to the acetylcholinesterase inhibitor (AChEI) donepezil, EGb 761 resulted in similar treatment outcomes over a 12-month period in patients with AD aged 80 or older while also having a more favorable safety profile [18]. In a cohort of 77 patients with vascular dementia who attended a memory clinic, treatment with EGb 761 alone or in combination with AChEI demonstrated cognitive and behavioural benefits after 12 months, as compared to AChEI monotherapy [19]. In more than 300 patients with prodromal dementia and AD followed at a dementia outpatient clinic for an average duration of 68 months, combination therapy of EGb 761 with AChEIs and/or memantine resulted in a slower progression as assessed by the Global Deterioration scale [20]. In an international multicentric non-interventional study involving more than 800 patients with mild to moderate AD, Mini-Mental State Examination (MMSE) scores after 12 months had improved with the combination of EGb 761 and AChEI, while they had deteriorated with AChEIs only [21]. The results of the present study add important information to this previous real-world data, as our data were collected from a large representative sample of physicians and patients that were followed for a long period of up to 10 years.

To date, no evidence has emerged to suggest that EGb 761 is more or less efficacious in specific forms of dementia. In both randomized clinical trials and real-world studies, the efficacy/effectiveness was comparable between dementia in Alzheimer’s disease and vascular [20,22]. The etiological diagnosis of cognitive impairment in routine care may be hindered by the lack of resources and/or limited access to biomarker assessment such as imaging. One advantage of EGb 761 is that it can be used independently of etiological diagnosis. Additionally, due to its favorable tolerability profile, it can be used safely in the early stages of the disease.

The progression rates of 12.7% in Gbe patients with mild or moderate dementia and 22.1% in non-Gbe patients with mild or moderate dementia are lower than expected. Davis et al. estimated annual transition probabilities to more severe stages of dementia for mild AD of 25% and for moderate AD of 36% [23]. This difference may be explained by the institutionalization of patients due to a worsening condition such as the transition to more severe stages of dementia, which results in a change in the attending physician. In this case, patients would be lost from our database. Moreover, deaths are often not reported to the office-based physicians and are, therefore, not added to the patients’ information in the database.

In Germany, drugs with Gbe as the active substance are available without prescription but are reimbursed by private and statutory health insurances if prescribed for the treatment of dementia. Reimbursement by the statutory health insurance requires a re-evaluation of treatment success after 12 weeks (Gemeinsamer Bundesausschuss (GBA) in Germany 2023) [24]. In addition, physicians might only prescribe Gbe to patients who they believe will benefit from it. The reasons for this assumption can be various, e.g., patients with an expected higher adherence due to their request of phytotherapeutics or less-adverse events compared to traditional antidementia drugs, patients with a mild course of disease, or patients with vascular dementia who cannot receive other antidementia drugs due to the lack of approval. This means that our analysis might have included a pre-selected group of patients who have a more favorable outcome.

The major strength of this study is the long follow-up period of up to 10 years, which allows for long-term, real-world assessment of Gbe in patients with dementia. Real-world data include diverse populations with few to no exclusion criteria, including patients with additional comorbidities, concomitant medications, and poor or unknown compliance, thereby increasing the generalizability of the results. Due to their longer duration, real-world studies show long-term treatment effects that are underestimated in randomized controlled trials and allow for the analysis of a variety of clinically relevant events, including rare events [25].

Furthermore, this is the first study to use a large dataset of qualitative information on degrees of severity based on the clinical judgement of physicians. Previous analyses in the real-world setting had to rely on proxies such as institutionalization of patients or prescription, as there is no documentation of these degrees by ICD codes. Regarding the use of clinical judgement, a multicenter prospective cohort study showed that the clinical judgement adds value to predicting dementia [26]. Therefore, it can be a valuable assessment of a patient’s degree of severity.

It should be noted, however, that the methodology employed in the study is not without certain limitations, which preclude the possibility of drawing causal inferences. Generalizability of results is limited by the different types of dementia included in the dataset. Moreover, incentives for entering dementia diagnoses into health records and reimbursement regulations varied during the study’s observation. These changes might have affected diagnosing dementia and prescribing behavior.

Another limitation is that the degrees of dementia severity are not based on formal ICD codes or on standardized assessments, such as the Mini-Mental State Examination (MMSE), Instrumental Activities of Daily Living (IADL), or a neurological examination with standardized documentation. Additionally, there is no information about the type of assessment used for the classification of severity, which may, therefore, be subjected to a certain variability and subjectivity.

Furthermore, the number of patients included in the analysis is rather low compared to the total number of mild and moderate dementia diagnosis in the IQVIA™ Disease Analyzer database. However, the study sample was sufficient to achieve adequate statistical power. The small study sample could be a result of the OTC sale of Gbe, which is not accounted for by the database since only prescriptions are recorded. Depending on the attitude of the prescribing physicians towards herbal medication and the treatment success of the initial period of 12 weeks, patients might not be able to obtain a Gbe prescription or reimbursement, respectively. This could lead to an increase in OTC purchases of Gbe, negatively influencing the frequency of repeat prescriptions recorded in the database. For the same reason, we were not able to analyze the duration of Gbe treatment.

A further limitation is the absence of data regarding the supportive measures taken by the patients, including the intake of dietary supplements, physical exercise, and cognitive training. These factors may act as confounders on the study results. Additionally, there is a lack of information on the patients’ socioeconomic status and their adherence to the Gbe treatment, which could have influenced the results. Both the missing information on adherence and the underreporting of Gbe use due to OTC sales can result in an underestimation of the effect of Gbe on the risk of dementia progression.

In conclusion, this study provides evidence from a real-world setting that Gbe prescription is associated with a reduced risk of dementia progression. These findings highlight the need for further research to explore the therapeutic potential of Gbe in long-term dementia management. Clinicians should consider the potential benefits of Gbe for patients with mild to moderate stages of dementia.

## Figures and Tables

**Table 1:** Baseline characteristics of patients.

Variable	Absolute Number (%) or Mean (SD)
N	4765
Age (mean, SD)	79.6 (6.5)
60–69 years (%)	1269 (26.6)
70–79 years (%)	1465 (30.8)
≥80 years (%)	2031 (42.6)
Sex: female (%)	3002 (63.0)
Sex: male (%)	1763 (37.0)
Statutory health insurance coverage (%)	4139 (86.9)
Private health insurance coverage (%)	626 (13.1)
Neuropsychiatric practice (%)	3046 (63.9)
General practice (%)	1719 (36.1)
Mild dementia (%)	2024 (42.5)
Moderate dementia (%)	2741 (57.5)
Depression (%)	1053 (22.1)
Parkinson’s disease (%)	277 (5.8)
History of stroke (%)	1024 (21.5)
Diabetes mellitus (%)	842 (17.7)
Hypertension (%)	1856 (39.0)
Ischemic heart disease or heart failure (%)	926 (19.4)
Lipid metabolism disorders (%)	768 (16.1)
Hearing impairment (%)	300 (6.3)
Prescription of antidementia drugs (%)	1027 (21.6)

**Table 2:** Frequency of Gbe prescription in patients with mild or moderate dementia.

Variable	Proportion of Patients with Gbe Prescription (N, %)
Total	177 (3.7)
60–69 years	50 (3.9)
70–79 years	64 (4.4)
≥80 years	63 (3.1)
Sex: female	104 (3.5)
Sex: male	73 (4.1)
Statutory health insurance coverage	129 (3.1)
Private health insurance coverage	48 (7.7)
Neuropsychiatric practice	54 (1.8)
General practice	123 (7.2)
Mild dementia	79 (3.9)
Moderate dementia	99 (3.6)

**Table 3:** Association between Gbe prescription and dementia progression (Cox regression models).

Cohort	Cumulative Incidence in Patients with Gbe Prescription (%)	Cumulative Incidence in Patients Without Gbe Prescription (%)	Crude Hazard Ratio (95% CI)	*p*-Value for Crude HR	Adjusted HR (95% CI) *	*p*-Value for Adjusted HR *
Total	12.7	22.1	0.35 (0.19–0.66)	0.001	0.50 (0.27–0.95)	0.033
Mild dementia	22.1	32.1	0.40 (0.20–0.81)	0.011	0.44 (0.22–0.90)	0.024
Moderate dementia	2.3	14.4	0.22 (0.05–0.87)	0.031	0.24 (0.06–0.98)	0.047

* Adjusted for age, sex, health insurance status, physician specialty, co-prescription of antidementia drugs, and co-diagnoses. HR—hazard ratio.

## Data Availability

The data presented in this study are available on request from the corresponding author. The data are not publicly available due to privacy restrictions.
